# Clinical–Biological Assessment of Prosthetic Field Following Pre-Prosthetic Phase Related to Prosthetic Treatment Solutions

**DOI:** 10.3390/clinpract15080140

**Published:** 2025-07-26

**Authors:** Petruţa Siminiuc, Doriana Agop-Forna, Cristina Dascălu, Norina Forna

**Affiliations:** Faculty of Dental Medicine, Grigore T. Popa University of Medicine and Pharmacy Iasi, Universitatii Street 16, 700115 Iasi, Romania; siminiucpetruta@yahoo.com (P.S.); cristina.dascalu@umfiasi.ro (C.D.); profforna@gmail.com (N.F.)

**Keywords:** clinical scores, stomatognathic system, preprosthetic procedures, tooth loss

## Abstract

**Background**. Extensive partial edentulism alters the biological and functional balance of the stomatognathic system, requiring targeted pre-prosthetic procedures to optimize treatment outcomes. **Objectives**. The aim of this study was to assess the extent of improvement in the clinical–biological scores of the prosthetic field in patients with extensive edentulism, following pre-prosthetic interventions. **Materials and Method**. This prospective, cross-sectional study investigated 194 subjects with extensive partial edentulism. Clinical–biological scores, initially and following the pre-prosthetic phase, were recorded using a scoring system that evaluated dental and periodontal status, bone and mucosal support, occlusion, and mandibulo-cranial relationships. Statistical comparisons of clinical–biological scores were related to the type of prosthetic therapy. Statistical significance was considered at a *p*-value < 0.05. **Results**. There was an overall significant improvement in the clinical–biological scores initially (mean value 20.2) and after pre-prosthetic procedures (mean value 23.22) (*p* < 0.001). When treatment groups were divided, the implant-assisted prosthesis group showed the best improvement in all domains, followed by the conventional fixed-prostheses group (*p* < 0.01). Dental support improved significantly in those with semi-rigid composite prostheses (*p* = 0.014), while periodontal support was improved in both fixed- and hybrid-implant groups. Mucosal and bone support improved mostly in the fixed-implant groups (*p* = 0.014). **Conclusions**. Pre-prosthetic procedures significantly enhance the biological and functional readiness of the prosthetic field, with the degree of improvement influenced by the complexity and type of planned prosthetic rehabilitation. The findings underscore the value of individualized pre-prosthetic protocols as an essential component of prosthetic treatment planning.

## 1. Introduction

Extensive partial edentulism continues to pose a complex challenge in prosthetic dentistry. The absence of multiple teeth in one or both arches often leads to profound biological and functional changes—affecting occlusion, periodontal health, bone integrity, and mandibulo-cranial relationships. Despite growing interest in the topic, the literature remains limited when it comes to quantifying the biological impact of pre-prosthetic interventions [1]. Preparation of the prosthetic field is intended to restore balance within the stomatognathic system by addressing dental, periodontal, mucosal, and bony deficiencies, while also correcting occlusal and functional discrepancies [2]. However, the degree to which these parameters improve, and how such improvements vary depending on the intended prosthetic solution, remains insufficiently documented.

Optimization of the clinical–biological parameters in the prosthetic field is necessary for the successful management of patients with extensive partial edentulism. An important part in gaining odontal support is the therapy of carious and non-carious lesions, and crown reshaping or resurfacing is a critical factor [3]. Concomitantly, periodontal support indices correlate significantly with non-surgical periodontal therapy, which may be accompanied by surgical options such as flap procedures, tissue grafts, or guided tissue regeneration in the post-prosthetic stage [4]. Mucosal and bony support restoration is generally performed with mucogingival grafting and bone augmentation and is essential for a more stable prosthetic outcome [5]. A correct diagnosis of occlusal and mandibulo-cranial discrepancies is of great importance in prosthetic planning, since this type of dysfunction can limit the possibilities of rehabilitation and affect final results if left uncorrected [6]. The restoration of mandibulo-cranial balance and overall function has been demonstrated with corrective measures, such as occlusal adjustment and transitional prostheses [7]. It is also very important to improve occlusal indices not only to ensure biomechanical health, but also to prevent esthetic limitations and malformations of denture perimeters [8]. The need for clinical–biological parameters as guiding elements in the planning of individualized prosthetic treatments has also been stressed in studies using expert systems for decision support in complex cases of partial edentulism [9]. Digital technologies have reshaped prosthetic dentistry by enhancing precision, efficiency, and personalization. Tools like digital impressions and CAD/CAM systems improve design accuracy and treatment predictability. As patients become more aware of these benefits—comfort, speed, and esthetics—digital workflows increasingly support both clinical success and patient expectations for modern care [10,11].

We intended to determine whether a scoring system developed at the Faculty of Dental Medicine (U.M.F. “Grigore T.Popa” Iasi) can offer an objective tool to assess the biological status of the prosthetic field and to consider adequate pre-prosthetic interventions according to local complexity.

*Null hypothesis:* there is no statistically significant improvement in the clinical–biological indices of the prosthetic field in patients with extensive partial edentulism following pre-prosthetic interventions, and these changes are not influenced by the planned prosthetic treatment modality.

*Aim of study:* to assess the extent of improvement in the clinical–biological indices of the prosthetic field and stomatognathic system components in patients with extensive partial edentulism requiring pre-prosthetic interventions, in relation to the selected prosthetic treatment strategy.

## 2. Materials and Methods

### 2.1. Study Design

In this prospective, cross-sectional study we evaluated patients with extensive partial edentulism, who were candidates for prosthetic therapy at the Clinical Base of the Dental Medicine Faculty, U.M.F. “Grigore T. Popa”, Iași.

This study adhered to the principles outlined in the Declaration of Helsinki and was approved by the Ethics Department of the University of Medicine and Pharmacy “Grigore T. Popa” (Nr. 444/23.05.2024) in Iași. All patients participating in the study provided informed consent by signing a consent form approved by the Ethics Committee of “Grigore T. Popa” University of Medicine and Pharmacy in Iași.

The study group included 194 patients (age: a mean age of 56.46 years ± 0.738 years and a range of 41–78 years; gender: 105 men and 89 women). The inclusion criteria were as follows: (i) aged over 18 years old, (ii) extensive partial edentulism in at least one arch, and (iii) eligible for fixed and removable prosthetic rehabilitation. The exclusion criteria were the following: complete edentulism, active oral infections, a recent history of maxillofacial trauma, and incomplete clinical or paraclinical documentation.

A priori power analysis was conducted with G*Power (version 3.1.9.7) to determine the minimum sample size necessary to detect a medium effect size (f = 0.25) in a repeated-measures analysis with two levels (pre and post intervention) based on the Wilcoxon signed-rank test, an alpha level of 0.05, and a power of 0.80 [12]. The required sample was 134 cases. Therefore our sample size of 194 patients exceeds the minimum requirement and ensures sufficient statistical power.

Eligible patients were randomly assigned to different treatment groups using a randomization algorithm to ensure balanced baseline characteristics and minimize allocation bias.

**Figure 1:**
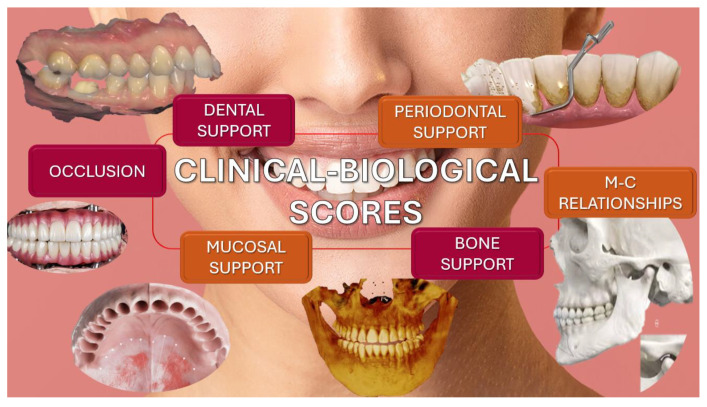
Clinical–biological scores used to assess prosthetic field status.

The evaluation of the prosthetic field status in the pre-treatment stage and after the pre-prosthetic phase was conducted by quantifying clinical–biological scores as follows: dental support; periodontal support; mucosal support; bone support; occlusion support; and mandibulo-cranial (M-C) relationships (Figure 1).

The clinical–biological scores were measured using the clinical scoring scale developed within the Discipline Extensive Partial Edentulism and Removable Restorations, Faculty of Dental Medicine, UMF “Grigore T. Popa”, Iași (Table 1). The criteria for measuring the quantitative scores are shown in Table 2, Table 3, Table 4, Table 5, Table 6 and Table 7. The clinical–biological scores were established based on the proportion of favorable criteria outlined in Table 2, Table 3, Table 4, Table 5, Table 6 and Table 7, with the aim of distinguishing degrees in a balanced and clinically meaningful manner. The “very good” score corresponds to an almost ideal level of support and was assigned to cases with at least 85% favorable criteria. The “good” scores reflect good, though not entirely optimal outcomes, including cases with 65% to 84% favorable criteria. The “medium” score applies to intermediate situations, with 40% to 64% favorable criteria, while the “low” score highlights cases with an unfavorable status, defined by less than 40% favorable criteria, which was considered insufficient for effective functional stability. The selection of these thresholds was based on the need to clearly differentiate clinically significant categories and to avoid excessively wide or inconsistent intervals.

The status of the prosthetic field and stomatognathic system components was evaluated at baseline (primary scores) and following the pre-prosthetic procedures (secondary scores). The criteria for selecting the materials used for the prostheses were closely linked to the clinical–biological condition of the prosthetic field and the type of prosthetic rehabilitation planned. The choice of prosthetic solution and materials was based on how well the patient’s condition matched key support parameters—dental, periodontal, mucosal, bone, occlusal, and mandibulo-cranial relationships—assessed both before and after pre-prosthetic interventions.

The scores were analyzed both at the level of group of the partially edentulous patients and in relation to prosthetic solutions (acrylic prosthesis; composite prosthesis with rigid special retention systems (SRMs); composite prosthesis with semi-rigid SRMs; elastic prosthesis; clasp-retained skeletal prosthesis; fixed implant-prosthetic (IP) rehabilitation; and hybrid removable IP rehabilitation).

### 2.2. Data Analysis

The statistical analysis was performed in SPSS 29.0. The qualitative data were reported as absolute values and percentages, and the quantitative data were reported as averages, standard deviations, standard errors, minimums, maximums, and medians. The Wilcoxon Z and Marginal Homogeneity Test were used to compare primary and secondary clinical–biological indices. *p*-values < 0.05 were evaluated as statistically significant, and *p*-values < 0.01 were evaluated as statistically highly significant.

## 3. Results

### 3.1. Changes in Prosthetic Field Scores Following Pre-Prosthetic Interventions in Patients with Extensive Partial Edentulism

In the total sample of patients with extensive edentulism, the mean score increased significantly from 20.2 in the primary assessment to 23.22 in the secondary assessment, with a Wilcoxon signed-rank test indicating a statistically significant difference (Z = −10,293, *p* < 0.001) (Table 8).

### 3.2. Changes in Stomatognathic System Scores Following Pre-Prosthetic Interventions According to Socio-Demographics Factors

**Figure 2:**
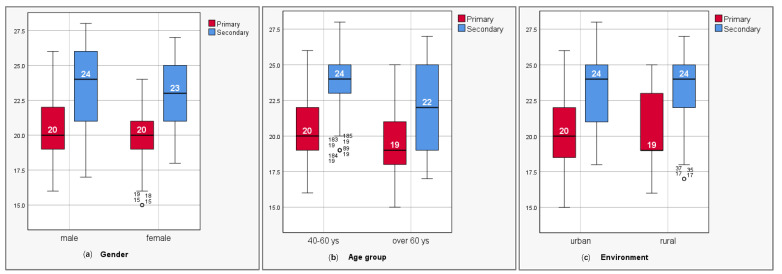
(**a**–**c**). Mean values of clinical–biological scores for gender (**a**), age group (**b**), and environment (residence) (**c**).

Table 9 presents the mean values of pre-operative clinical–biological indices (primary) and those after the end of pre-prosthetic interventions (secondary), related to socio-demographic factors. The comparative analysis between the primary and secondary evaluation phases revealed significant improvements in mean scores across all socio-demographic groups. The highest increase was observed in the 40–60 yr. age group, where the mean score rose from 20.61 to 23.80, indicating an improvement of 3.19 points. This was followed by the female group, which recorded an increase of 3.16 points (from 19.90 to 23.06), and the urban group, with an improvement of 3.07 points (from 20.20 to 23.27). In the total sample, the mean score increased from 20.20 to 23.22, resulting in a 3.02 point gain. The male group registered a slightly lower increase of 2.89 points, from 20.46 to 23.35. The smallest improvement was observed in the group aged over 60 yrs., with an increase of 2.72 points, from 19.47 to 22.19. These results suggest that although all socio-demographic groups benefited from the pre-prosthetic interventions, with highly statistically significant differences (*p* < 0.001 **), the 40–60 yr. age group and female patients showed the highest degree of positive changes regarding the status improvement in the stomatognathic system components (Table 9; Figure 2a–c).

### 3.3. Changes in Prosthetic Field Scores Following Pre-Prosthetic Interventions According to Planned Prosthetic Treatment Solution

**Figure 3:**
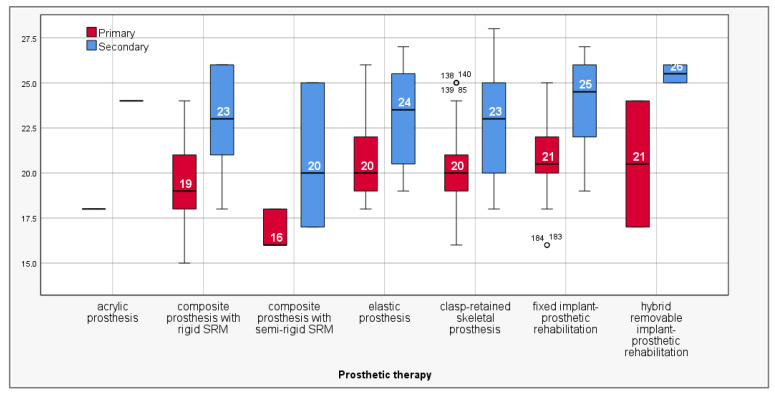
Mean values of primary and secondary clinical–biological scores related to prosthetic solutions.

Table 10 presents the mean values of pre-operative clinical–biological indices (primary) and those after the end of pre-prosthetic interventions (secondary), related to prosthetic treatment modalities. The analysis of clinical–biological score improvements based on the prosthetic treatment modality revealed notable differences in treatment outcomes. Patients eligible for acrylic prosthesis treatment recorded the highest increase in mean score, with an improvement of 6.00 points (from 18.00 to 24.00), although this result did not reach statistical significance (*p* = 0.157). Patients eligible for removable hybrid implant-prosthetic rehabilitation showed statistically significant improvement (*p* = 0.024) with a gain of 5.00 points (from 20.50 to 25.50). Patients treated with composite prostheses with semi-rigid special retention systems (SRSs) had a mean score increase of 4.00 points (from 16.67 to 20.67), with statistical significance (*p* = 0.007). Patients eligible for composite prostheses with rigid special retention systems (SRSs) showed an improvement of 3.61 points (from 19.35 to 22.96). Patients suited for fixed implant-prosthetic rehabilitation reached a mean increase of 3.29 points (from 20.71 to 24.00), a highly significant statistical result (*p* < 0.001). Patients eligible for elastic prostheses and clasp-retained skeletal prostheses also experienced highly statistical significant improvements (*p* < 0.001 **), with increases of 2.50 and 2.54 points, respectively (Table 10; Figure 3).

In the group of patients rehabilitated with acrylic prostheses, the clinical–biological scores showed no variability between the initial and secondary assessments, maintaining consistent values across all parameters. Dental support and bone support were rated as “good” in both the primary and secondary phases. Periodontal support also remained at a “very good” level throughout the evaluation. Mucosal support was classified as “medium” for both patients in both assessments. Occlusal support and mandibulo-cranial relationships, initially rated as “low,” were both reclassified as “very good” after the completion of the pre-prosthetic phase. The total score improved accordingly, shifting from “good” in the primary assessment to “very good” in the secondary evaluation, indicating a favorable clinical evolution following pre-prosthetic interventions (Table 11).

In the group of patients rehabilitated with composite prostheses using rigid SRMs, several clinically significant improvements were observed following pre-prosthetic interventions. Dental support showed a marked improvement from 42.3% of patients rated as “medium”, 42.3% as “good,” and 15.4% as “very good” to only 7.7% at the “medium” level, while 53.8% reached the “very good” scores (*p* < 0.001) after the pre-prosthetic stage. Periodontal support improved from 61.5% rated as “good” and 38.5% as “very good” in the initial stage to 100% “very good” post intervention. Scores of mucosal support increased with the proportion of patients rated as “very good” increasing from 15.4% to 84.6% (*p* = 0.025). Bone support scores changed from 26.9% “very good” to 42.3% after the pre-prosthetic interventions (*p* = 0.013). Occlusion scores improved notably, with “low” scores dropping from 88.5% to 46.2%, while the “very good” category increased from 0% to 46.2% (*p* < 0.001). Mandibulo-cranial relationships increased from 23,1% “very good” scores initially to 34.6% post intervention (*p* = 0.041). Overall, the total score distribution shifted from 80.8% of patients rated as “good” to 46.2% with “very good” scores and 46.2% with “good” scores after pre-prosthetic interventions (Table 12).

In the group of patients rehabilitated with composite prostheses using SRMs, improvements were observed across several clinical parameters following the pre-prosthetic interventions. Regarding dental support, a statistically significant improvement was noted (*p* = 0.014). Initially, 66.7% of patients were rated as having “good” scores and 33.3% as “very good”. After the pre-prosthetic intervention, the proportion of “very good” scores increased to 33.3%. Periodontal support showed an upward trend, although not statistically significant (*p* = 0.083). At baseline, the scores were evenly distributed (33.3%) across “medium”, “good”, and “very good” categories. Following the intervention, 66.7% of patients were rated as “good” and one-third (33.3%) as “very good”. Initially, 66.7% of patients had “good” mucosal support and 33.3% “medium”, but post intervention, all patients (100%) were rated as “good”. Bone support improved from 66.7% “good” and 33.3% “medium” to 100% “good”. In terms of occlusion, there was a positive change, although not statistically significant (*p* = 0.083). While all patients were initially rated as “low,” this score decreased to 66.7% post intervention, while 33.3% reached the “very good” category. Mandibulo-cranial relationships demonstrated a statistically significant improvement (*p* = 0.028), with the initial 100% of patients rated as “low” shifting to 33.3% each across “low”, “medium”, and “very good” categories after the pre-prosthetic phase. The total scores also showed a statistically significant change (*p* = 0.020), improving from 100% of patients rated as “good” at baseline to a balanced distribution of 33.3% for “medium”, “good”, and “very good” after the pre-prosthetic phase (Table 13).

In the group of patients rehabilitated with elastic prostheses, the pre-prosthetic interventions led to consistent clinical improvements across several functional and anatomical parameters. Initially, 83.3% of patients were rated as having a “very good” general condition, and 16.7% as “good”. After intervention, the percentage of “very good” scores increased to 91.7%, with only 8.3% remaining at the “good” level. Dental support demonstrated a statistically significant improvement (*p* < 0.001). Before the intervention, 25.0% of patients were rated as “medium”, 66.7% as “good”, and 8.3% as “very good”. After the pre-prosthetic phase, 41.7% of patients achieved “very good” scores, 50.0% remained at “good”, and only 8.3% were still at the “medium” level. Periodontal support also improved significantly (*p* = 0.014). Initially, 8.3% of patients were rated as “medium”, 16.7% as “good”, and 75.0% as “very good”. Following the pre-prosthetic interventions, 83.3% reached the “very good” level, while 16.7% remained at “good”. Mucosal support remained unchanged (*p* = 1.000). Two-thirds of the patients (66.7%) were consistently rated as having “good” mucosal support, and one-third (33.3%) as “very good”, both before and after the intervention. Bone support showed a slight, non-significant improvement (*p* = 0.083). Initially, 8.3% of patients were rated as “medium”, 50.0% as “good”, and 41.7% as “very good”. After the pre-prosthetic phase, 50.0% of patients were rated as “very good”, 41.7% as “good”, and 8.3% as “medium”, indicating a positive, though modest, shift. Occlusion scores improved significantly (*p* < 0.001). At baseline, 75.0% of patients had “low” occlusion scores, 16.7% “good”, and 8.3% “medium”. After intervention, the “low” category dropped to 41.7%, while 25.0% of patients reached the “very good” level, 25.0% were rated “good”, and 8.3% remained at “medium”. Mandibulo-cranial relationships also showed a significant enhancement (*p* = 0.003). Initially, half of the patients (50.0%) were rated as “low”, 33.3% as “very good”, and 8.3% each as “medium” and “good”. Post intervention, 58.3% of patients reached “very good”, 25.0% remained “low”, and 8.3% were rated “medium” and “good”, respectively. The total score showed a statistically significant improvement (*p* = 0.001). At baseline, most patients (75.0%) were rated as “good”, 16.7% as “medium”, and only 8.3% as “very good”. After the pre-prosthetic phase, 41.7% of patients were rated as “very good”, 50.0% as “good”, and only 8.3% remained at “medium” (Table 14).

In the group of patients rehabilitated with clasp-retained skeletal prostheses, significant improvements were observed following the pre-prosthetic interventions in most clinical parameters evaluated. Dental support showed a highly significant improvement (*p* < 0.001). Initially, 31.5% of patients were rated as “medium”, 58.9% as “good”, and only 9.6% as “very good”. After the pre-prosthetic interventions, the proportion of patients in the “very good” category rose substantially to 43.8%, while “good” ratings dropped to 42.5% and “medium” to 13.7%. Periodontal support also improved significantly (*p* < 0.001). At baseline, 5.5% of patients were rated as “medium”, 35.6% as “good”, and 58.9% as “very good”. Following the pre-prosthetic interventions, 82.2% of patients were in the “very good” category, and 17.8% were rated as “good”. Mucosal support showed a statistically significant change as well (*p* < 0.001). Initially, 8.2% of patients were rated as “medium”, 69.9% as “good”, and 21.9% as “very good”. After the pre-prosthetic interventions, 31.5% of patients were in the “very good” category, and 68.5% were rated as “good”. Bone support exhibited a modest, non-significant improvement (*p* = 0.083). At baseline, 4.1% of patients were rated as “medium”, 43.8% as “good”, and 52.1% as “very good”. After the pre-prosthetic interventions, 56.2% were in the “very good” category, 39.7% “good”, and 4.1% remained “medium”. Occlusion scores improved markedly and significantly (*p* < 0.001). Initially, 82.2% of patients were rated as having “low” occlusion, while only 4.1% each were rated as “good” and “very good”. Post intervention, the “low” category decreased to 52.1%, with 17.8% as rated “good” and 26.0% as “very good”. Mandibulo-cranial relationships also demonstrated significant improvement (*p* < 0.001). At baseline, 52.1% of patients were rated as “low”, 34.2% as “very good”, and the remaining were divided between “medium” and “good”. After the pre-prosthetic interventions, the proportion of patients rated as “very good” rose to 56.2%, while “low” dropped to 31.5%. The total score showed a highly significant improvement (*p* < 0.001). Initially, 24.7% of patients were rated as “medium”, 67.1% as “good”, and only 8.2% as “very good”. After the pre-prosthetic interventions, 39.7% of patients reached the “very good” category, 52.1% were rated as “good”, and only 8.2% remained “medium” (Table 15).

In the group of patients undergoing fixed implant-prosthetic rehabilitation, the pre-prosthetic interventions resulted in statistically significant improvements across nearly all evaluated clinical parameters. Dental support improved significantly (*p* < 0.001). Initially, 14.3% of patients were rated as “medium”, 71.4% as “good”, and 14.3% as “very good”. After the pre-prosthetic interventions, the proportion of patients rated as “very good” increased to 42.9%, while “good” decreased to 57.1%, and “medium” ratings were no longer present. Periodontal support also showed significant improvement (*p* < 0.001). At baseline, 14.3% of patients were rated as “medium”, 28.6% as “good”, and 57.1% as “very good”. After the pre-prosthetic interventions, the “very good” category rose to 78.6%, with 14.3% rated as “good” and only 7.1% remaining at the “medium” level. Mucosal support showed a statistically significant shift (*p* = 0.005). Initially, 14.3% of patients were rated as “medium”, 35.7% as “good”, and 50.0% as “very good”. Post intervention, 64.3% of patients were rated as “very good” and 35.7% as “good”, with no patients remaining in the “medium” category. Bone support improved significantly (*p* = 0.014). At the beginning, 14.3% of patients were rated as “medium”, 57.1% as “good”, and 28.6% as “very good”. After the pre-prosthetic interventions, 42.9% were rated as “very good”, 42.9% as “good”, and 14.3% remained “medium”. Occlusion showed one of the most substantial improvements (*p* < 0.001). Initially, 85.7% of patients had “low” occlusion ratings, while 7.1% each were rated as “medium” and “good”. Following the pre-prosthetic interventions, the “low” category dropped to 35.7%, and 42.9% of patients reached the “very good” level. Additionally, 14.3% were rated as “good” and 7.1% as “medium”. Mandibulo-cranial relationships also improved significantly (*p* < 0.001). At baseline, 35.7% of patients were rated as “low”, 42.9% as “very good”, and the remaining were distributed between “medium” (7.1%) and “good” (14.3%). After the pre-prosthetic interventions, 71.4% of patients reached the “very good” category, while “low” ratings decreased to 14.3%. The total score demonstrated a highly significant improvement (*p* < 0.001). Initially, most patients (78.6%) were rated “good”, 14.3% as “very good”, and 7.1% as “medium”. Following the pre-prosthetic interventions, the scores were evenly split: 50.0% of patients were rated as “very good” and 50.0% as “good”, with no patients remaining at the “medium” level (Table 16).

In the group of patients rehabilitated with removable hybrid implant-prosthetic restorations, clinical improvements were observed across several parameters following pre-prosthetic interventions. Dental support remained stable, with all patients (100%) rated as having “good” support both pre and post intervention. Periodontal support showed improvement, although not tested for statistical significance. Initially, half of the patients (50.0%) were rated as “good” and the other half as “very good”. After the pre-prosthetic intervention, 100% of the patients were rated as having “very good” periodontal support. Mucosal support remained unchanged (*p* = 1.000), with 50.0% of patients rated as “good” and 50.0% as “very good” at both evaluation stages. Bone support also showed no statistical difference (*p* = 1.000). At both time points, half of the patients (50.0%) were rated as having “medium” bone support and half as “very good”. Occlusion scores improved significantly (*p* = 0.028). Initially, all patients (100%) were rated as having “low” occlusion. After the pre-prosthetic interventions, scores shifted evenly, with 50.0% rated as “medium” and 50.0% as “very good”. Mandibulo-cranial relationships showed a clear improvement, though without statistical testing. At baseline, 50.0% of patients were rated as “low” and 50.0% as “very good”. Following the pre-prosthetic intervention, 100% of patients were rated as “very good”. The total score also improved, with a shift from 50.0% “good” and 50.0% “very good” before intervention to 100% “very good” after the pre-prosthetic phase (Table 17).

## 4. Discussion

### 4.1. Influence of Pre-Prosthetic Interventions on Outcome of Prosthetic Treatment in Extensive Edentulism

The status of muco-osseous support and other stomatognathic systems components influence significantly temporo-mandibular joint status, which represents a mechanically demanding environment [13,14,15]. Edentulism has increasingly been recognized as a major public health issue, particularly given the estimate that in 2030, persons aged 65 and older will make up 26% of the population [14,15]. At the same time, alveolar bone resorption and residual ridge irregularity produced by extensive partial edentulism will affect occlusion and cranio-mandibular relationships, which have a direct relationship with temporomandibular joint (TMJ) disorders [8]. Such complications may comprise dental migrations, tooth extrusions, facial modification, and/or muscle or joint dysfunction [13,14,15]. Successful prosthetic rehabilitation in such patients would require a team approach, especially considering regional and systemic factors. Local conditions, such as the number and distribution of remaining teeth, residual ridge, mucosal and osseous support, occlusion, and cranio-mandibular relationship must be thoroughly assessed [16]. At the systemic level, neuromuscular control, psychological status, overall health robustness, and even economic limitations or patient preference contribute to treatment planning [17]. Both pre-prosthetic interventions and pro-prosthetic management are necessary to maintain biomechanical integrity. These phases are intended to modify the negative clinical and biological indices of the prosthetic field in view of the functional interdependence of the components of the stomatognathic system [18]. A prosthetic treatment should therefore aim to restore and harmonize all structures of the muco-osseous and functional systems, and complications such as malocclusion, mandibular deviation, discomfort in the TMJ, and alteration of the cranio-mandibular coordination should be resolved [17,18,19]. Pre-prosthetic interventions should be directed toward possible etiological factors from various aspects, requiring the clinician to have a comprehensive knowledge of the functional, anatomical, and biological conditions of the prosthetic field [20].

In this context, we used a quantitative score developed at the Faculty of Dental Medicine, UMF “Grigore T. Popa”, Iasi (Romania), to investigate the scale of modifications in the stomatognathic system components following intervention in the pre-prosthetic phase. The extended and detailed interpretation of the changes in clinical–biological scores among different patient categories highlights not only the varying magnitude of improvements depending on the type of prosthetic treatment but also the differentiated need for pre-prosthetic interventions to ensure a biologically and functionally adequate prosthetic field. Patients rehabilitated with acrylic prostheses showed the highest increase in mean scores (+6.00 points), although without statistical significance. Although the group of patients rehabilitated with acrylic prostheses showed the highest numerical increase in clinical–biological scores following pre-prosthetic interventions (+6.00 points), this change did not reach statistical significance. However, a qualitative examination of the results revealed substantial changes in occlusal support and mandibulo-cranial relationships, both of which progressed from a “low” to a “very good” classification after pre-prosthetic treatment. These findings highlight the particular need for functional correction and occlusal rebalancing in patients receiving mucosa-borne prosthetic solutions. Even in a small sample, the magnitude of the clinical–biological score improvement supports the idea that pre-prosthetic interventions are indispensable for achieving optimal outcomes in removable mucosa-supported prostheses. Further research with larger cohorts is necessary to confirm these trends and explore whether these improvements translate into long-term prosthetic success and patient satisfaction. Since acrylic dentures rely on mucosal support, they demand significant modifications in the prosthetic field—through occlusal rebalancing, mucosal treatments, or planned rebasing—which justifies the significant changes in the clinical–biological indices [21]. In contrast, patients treated with removable hybrid implant-supported prostheses achieved a statistically significant improvement (+5.00 points, *p* = 0.024), with uniform progress across nearly all components of the stomatognathic system. These results are explained by the dual nature of the prosthesis—implant-supported but removable—which requires both biomechanical stabilization of cranio-mandibular relations and conditioning of the prosthetic field (bone support, occlusion, and periodontal balance) [22,23,24]. The necessary pre-prosthetic interventions are therefore more complex and multidisciplinary, which justifies the substantial score changes. Patients treated with composite prostheses using rigid retention systems showed an average improvement of 3.61 points, while those with semi-rigid systems achieved a 4.00 point increase (*p* = 0.007). In both cases, the score changes reflect the need to correct dental, periodontal, and occlusal balance to ensure optimal integration of the mechanical retention components. Rigid systems require a more precise clinical–biological adaptation, especially in terms of bone and periodontal support, to prevent overloading and maintain long-term function. Therefore, pre-prosthetic interventions in these cases are relatively complex but targeted, which explains the moderate yet significant score increases [25]. Patients rehabilitated with elastic prostheses showed a score increase of 2.50 points (*p* < 0.001), while those with clasp-retained skeletal prostheses recorded a similar increase of 2.54 points. These less invasive prosthetic solutions involve functional adaptation of the prosthetic field that does not require extensive biological or surgical interventions. For elastic prostheses, improvements are primarily related to occlusal balance and functional relationships due to the prosthesis’ flexibility and mucosal resilience. In the case of skeletal prostheses, the most notable progress occurred in cranio-mandibular relationships and occlusion, indicating that functional rebalancing rather than biological intervention is the main requirement [26]. Fixed implant-supported rehabilitations led to a mean increase of 3.29 points (*p* < 0.001), with significant progress across all evaluated parameters.

These results underline the major importance of pre-prosthetic stages in these cases, as the long-term success of fixed prostheses depends on a perfectly balanced biological environment.

Pre-prosthetic procedures are rigorous and multidimensional—addressing periodontal, surgical, functional, and intermaxillary aspects. As such, even if the score increase is not the highest, it is consistent and broadly distributed across the entire stomatognathic system. In comparison, acrylic and hybrid implant-supported prostheses require the most extensive pre-rehabilitation corrections, justifying the highest score increases [26,27]. In contrast, treatments using special retention systems (rigid or semi-rigid) involve more focused biomechanical and biological adaptations, while skeletal and elastic prostheses require more conservative, functionally oriented approaches with smaller but significant score variations. Fixed implant-supported rehabilitations, despite more moderate score changes, stand out due to the coherence of improvements and the high standard of biological correction required, reflecting rigorous and multidisciplinary clinical planning [28,29,30,31,32,33].

These differences confirm that the magnitude of clinical–biological score improvements is directly related to the complexity and type of prosthetic solution planned: the more the rehabilitation relies on long-term functional stability, the more a thoroughly balanced prosthetic field is required, and the more elaborate and decisive the pre- and pro-prosthetic interventions must be to ensure the success of treatment.

### 4.2. Practical Implications

This research highlights the complications of extensive partial edentulism and the need for proper quantification of the improvements obtained in the pre-prosthetic stage to optimize treatment protocols, minimize failure risks, and ensure long-term functional and esthetic outcomes in complex fixed and removable prosthetic rehabilitations. The therapeutic management of the patients with extensive partial edentulism requires both theoretical and practical knowledge of the optimal parameters of stomatognathic system components to avoid biomechanical instability, esthetics failure, and improper design of the future prosthetic restorations.

### 4.3. Limitations

The current study has a number of limitations that need to be considered. First, the observational and non-randomized design hinders the possibility of establishing a cause–effect relationship between pre-prosthetic treatments and variations in the clinical–biological scores of the prosthetic field. A further limitation was the selection of the sample from a single clinical context and the potential impact that this would have on the generalizability to other populations with varying socio-economic, geographic, or healthcare access characteristics. Although socio-demographic factors were taken into consideration, other potential confounders (systemic health status, medication consumption, parafunctional habits, and previous prosthetic experience) were not adequately controlled and might have introduced some biases in the results. Furthermore, some treatment subgroups (i.e., the group rehabilitated with an acrylic prosthesis or implant-supported removable rehabilitation) were represented by only a small patient sample, which reduced the clinical trial’s statistical power. The lack of objective functional evaluation (such as electromyography, analysis of occlusal force, or more detailed tests of masticatory efficiency) may have led to an underestimation of clinical effects, especially in borderline and subclinical cases. The short period of follow-up does not make it possible to determine whether the prosthetic treatments performed are stable and durable in the longer term. The method of anatomic and functional assessment was not as accurate as might be achieved by modern objective methods such as 3D imaging or digital occlusal analysis. Furthermore, the absence of patient-reported outcomes (quality of life or satisfaction) makes it impossible to correlate the biological scores with the real patient experience.

### 4.4. Future Research Directions

Digital previews of prosthetic outcomes contribute to greater patient involvement by offering enhanced transparency and fostering trust in the proposed treatment plan [34,35]. As patients become more familiar with these technologies, the demand for digitally assisted prosthetic solutions is likely to rise. Further research is needed to investigate how integrating digital workflows into the pre-prosthetic phase impacts both clinical performance and patient satisfaction. As future perspectives, objective digital evaluations (CBCT, 3D scans, and computerized occlusal analysis) should be included to more objectively validate the biological and functional modifications of the prosthetic field. Long-term studies are necessary to investigate the durability of clinical and biological enhancements and to correlate these with prosthetic success. Multihospital and randomized study designs would increase external validity and provide standardized pre-prosthetic protocols. Incorporating self-reported outcomes, for example, perceived masticatory efficiency and comfort, should allow assessment of treatment efficacy from another perspective. Additionally, the possibility of artificial intelligence tools being used in personalized prosthetic planning could be investigated in further studies.

## 5. Conclusions

The efficiency of a pre-prosthetic treatment is directly related to the type of prosthetic rehabilitation to be performed. These cases demand a more extensive adjustment of occlusion and cranio-mandibular relationships, revealing the relevance of thorough preparation in cases with greater prosthetic complexity. Patients eligible for fixed implant-supported and fixed composite prostheses (rigid and semi-rigid retention) provided strong evidence of statistically significant improvement in all stomatognathic system functional components, allowing a balance between the biological and biomechanical condition before definitive prosthetic management. In patients eligible for elastic and clasp-retained skeletal prostheses, the enhancement in prosthetic field scores was generally moderate, with significant changes especially in occlusal and mandibulo-cranial relationships. The amount of clinical–biological score improvement indicates the functional needs and biomechanical properties for each prosthetic concept, requiring tailored pre-prosthetic procedures. These protocols should further be adjusted to the expected distribution of loading, retention system, and anatomical complexity of the prosthetic field to optimize the treatments especially in partial extensive edentulism.

## Figures and Tables

**Table 1:** Quantitative scores of prosthetic field and stomatognathic system components.

SCORE	Score 1	Score 2	Score 3	Score 4
STATUS	Low	Medium	Good	Very good
Percentage of favorable criteria	<40%	40–64%	65–84%	>85%

**Table 2:** Favorable and unfavorable criteria scores of dental support.

*Favorable criteria*	*Unfavorable criteria*
Absence of dental anomalies	Presence of dental anomalies
Normal or surface contact points	Absent contact points
No abrasion	Presence of abrasion
No carious lesions	Presence of carious lesions
No coronal restorations	Presence of coronal restorations
No endodontic complications	Presence of endodontic complications
Tooth distribution on arch (4 quadrants)	Tooth distribution on arch (1–3 quadrants)
Number of teeth present (9–14)	Number of teeth present (<9)

**Table 3:** Favorable and unfavorable criteria scores of periodontal support.

*Favorable criteria*	*Unfavorable criteria*
Absence of interdental papilla bleeding	Presence of interdental papilla bleeding
Absence of gingival recession	Presence of gingival recession
Absence of interradicular lesion	Presence of interradicular lesion
Absence of tooth mobility	Presence of tooth mobility

**Table 4:** Favorable and unfavorable criteria scores of occlusal support.

*Favorable criteria*	*Unfavorable criteria*
Normal occlusal areas	Modified occlusal areas
Normal supporting cusps	Modified supporting cusps
Normal guiding cusps	Modified guiding cusps
Normal grooves, fossae, embrasures	Modified grooves, fossae, embrasures
Normal retroincisal slope	Modified retroincisal slope
Normal sagittal occlusal curve	Modified sagittal occlusal curve
Normal transverse occlusal curve	Modified transverse occlusal curve
Normal frontal curvature	Modified frontal curvature
Normal occlusal plane	Modified occlusal plane
Normal centric relation closure path	Modified centric relation closure path
Normal posture relation closure path	Modified posture relation closure path
Normal lateral movements	Modified lateral movements
Normal protrusion	Normal protrusion

**Table 5:** Favorable and unfavorable criteria scores of bone support.

*Favorable criteria*	*Unfavorable criteria*
Intact alveolar arch Mx/Md	Modified alveolar arch Mx/Md
Mx tuberosity—normal shape and retention	Mx tuberosity—modified shape and retention
Pyriform tubercle—normal shape and retention	Pyriform tubercle—modified shape and retention
Palatal vault—symmetrical, medium depth	Palatal vault—asymmetrical, flat depth
Absent palatal torus	Present palatal torus
Absent mandibular torus	Present mandibular torus

**Table 6:** Favorable and unfavorable criteria scores of mucosal support.

*Favorable criteria*	*Unfavorable criteria*
Alveolo-jugal fold insertion > 4 mm	Alveolo-jugal fold insertion of 2–4 mm or <2 mm
Labial frenum insertion > 4 mm	Labial frenum insertion of 2–4 mm or <2 mm
Normal functional areas Mx/Md	Shortened/interrupted functional areas
Low lingual floor	Medium/high lingual floor
Mx tuberosity mucosa—normal	Mx tuberosity mucosa—increased
Pyriform tubercle mucosa—normal	Pyriform tubercle mucosa—increased
Healthy fibromucosa	Inflamed fibromucosa
Fibromucosa with normal resilience	Fibromucosa with increased resilience

**Table 7:** Favorable and unfavorable criteria scores of mandibulo-cranial relationship.

*Favorable criteria*	*Unfavorable criteria*
Relationship of posture (TMJ reference)—normal	Relationship of posture (TMJ reference)—modified
Relationship of posture (mandibular reference)—normal	Relationship of posture (mandibular reference)—modified
Relationship of posture (dental reference)—normal	Relationship of posture (dental reference)—modified
Relationship of posture (bone reference)—normal	Relationship of posture (bone reference)—modified
Relationship of posture (labial reference)—normal	Relationship of posture (labial reference)—modified
Relationship of posture (TMJ reference)—normal	Relationship of posture (TMJ reference)—modified
Relationship of posture (mandibular reference)—normal	Relationship of posture (mandibular reference)—modified
Relationship of posture (dental reference)—normal	Relationship of posture (dental reference)—normal
Relationship of posture (bone reference)—normal	Relationship of posture (bone reference)—normal
Relationship of posture (labial reference)—normal	Relationship of posture (labial reference)—normal

**Table 8:** Clinical–biological score changes in study group.

Group	Phase	N	Mean (CI 95%)	Std. Error	Std. Deviation	Min	Max	Median (IQR)	Wilcoxon Z	*p*-Value
Total	Primary scores	194	20.20 (19.85 ÷ 20.56)	0.18	2.507	15	26	20.00 (19.00 ÷ 22.00)	Z = −10.293	*p* < 0.001 **
	Secondary scores	194	23.22 (22.83 ÷ 23.60)	0.197	2.742	17	28	24.00 (21.00 ÷ 25.00)		

**: Highly statistical significant.

**Table 9:** Primary and secondary scores (mean values) following the pre-prosthetic stage in patients with extensive partial edentulism: socio-demographic parameters.

Group	Phase	N	Mean (CI 95%)	Std. Error	Std. Deviation	Min	Max	Median (IQR)	Wilcoxon Z	*p*-Value
Male	Primary	105	20.46 (19.93 ÷ 20.99)	0.267	2.739	16	26	20.0 (19.0 ÷ 22.5)	Z = −7.265	*p* < 0.001 **
	Secondary	105	23.35 (22.80 ÷ 23.91)	0.28	2.872	17	28	24.0 (21.0 ÷ 26.0)		
Female	Primary	89	19.9 (19.44 ÷ 20.36)	0.231	2.18	15	24	20.0 (18.5 ÷ 21.0)	Z = −7.368	*p* < 0.001 **
	Secondary	89	23.06 (22.51 ÷ 23.60)	0.274	2.587	18	27	23.0 (20.5 ÷ 25.0)		
Age 40–60	Primary	124	20.61 (20.16 ÷ 21.06)	0.228	2.533	16	26	20.0 (19.0 ÷ 22.0)	Z = −8.707	*p* < 0.001 **
	Secondary	124	23.8 (23.38 ÷ 24.21)	0.209	2.327	19	28	24.0 (23.0 ÷ 25.0)		
Age >60	Primary	70	19.47 (18.92 ÷ 20.02)	0.275	2.301	15	25	19.0 (18.0 ÷ 21.0)	Z = −5.532	*p* < 0.001 **
	Secondary	70	22.19 (21.44 ÷ 22.93)	0.372	3.113	17	27	22.0 (19.0 ÷ 25.0)		
Urban	Primary	148	20.2 (19.81 ÷ 20.60)	0.199	2.419	15	26	20.0 (18.25÷22.0)	Z = −9.216	*p* < 0.001 **
	Secondary	148	23.27 (22.84 ÷ 23.70)	0.219	2.666	18	28	24.0 (21.0 ÷ 25.0)		
Rural	Primary	46	20.2 (19.36 ÷ 21.03)	0.413	2.802	16	25	19.0 (19.0 ÷ 23.0)	Z = −4.634	*p* < 0.001 **
	Secondary	46	23.04 (22.15 ÷ 23.93)	0.442	2.996	17	27	24.0 (21.25÷25.0)		

**: Highly statistical significant.

**Table 10:** Primary and secondary scores in patients with extensive partial edentulism: prosthetic treatment modality.

Group	Phase	N	Mean (CI 95%)	Std. Error	Std. Deviation	Min	Max	Median (IQR)	Wilcoxon Z	*p*-Value
Acrylic prosthesis	Primary	2	18.0 (18.00 ÷ 18.00)	0.0	0.0	18	18	18.0 (18.0 ÷ 18.0)	Z = −1.414	*p* = 0.157
	Secondary	2	24.0 (24.00 ÷ 24.00)	0.0	0.0	24	24	24.0 (24.0 ÷ 24.0)		
Composite prosthesis with rigid SRM	Primary	26	19.35 (18.28 ÷ 20.41)	0.517	2.637	15	24	19.0 (18.0 ÷ 21.0)	Z = −4.318	*p* < 0.001 **
	Secondary	26	22.96 (21.83 ÷ 24.09)	0.548	2.793	18	26	23.0 (20.75 ÷ 26.0)		
Composite prosthesis with semi-rigid SRM	Primary	9	16.67 (15.90 ÷ 17.44)	0.333	1.0	16	18	16.0 (16.0 ÷ 18.0)	Z = −2.694	*p* = 0.007 **
	Secondary	9	20.67 (17.98 ÷ 23.36)	1.167	3.5	17	25	20.0 (17.0 ÷ 25.0)		
Elastic prosthesis	Primary	36	20.67 (19.92 ÷ 21.41)	0.367	2.204	18	26	20.0 (19.0 ÷ 22.0)	Z = −4.323	*p* < 0.001 **
	Secondary	36	23.17 (22.22 ÷ 24.12)	0.467	2.803	19	27	23.5 (20.25÷ 25.75)		
Clasp-retained skeletal prosthesis	Primary	73	20.45 (19.90 ÷ 21.00)	0.275	2.351	16	25	20.0 (19.0 ÷ 21.0)	Z = −5.664	*p* < 0.001 **
	Secondary	73	22.99 (22.35 ÷ 23.63)	0.321	2.746	18	28	23.0 (20.0 ÷ 25.0)		
Fixed implant-prosthetic rehabilitation	Primary	42	20.71 (19.98 ÷ 21.44)	0.361	2.34	16	25	20.5 (20.0 ÷ 22.0)	Z = −5.038	*p* < 0.001 **
	Secondary	42	24.0 (23.28 ÷ 24.72)	0.354	2.295	19	27	24.5 (22.0 ÷ 26.0)		
Removable hybrid implant-prosthetic rehabilitation	Primary	6	20.5 (16.48 ÷ 24.52)	1.565	3.834	17	24	20.5 (17.0 ÷ 24.0)	Z = −2.251	*p* = 0.024 *
	Secondary	6	25.5 (24.93 ÷ 26.07)	0.224	0.548	25	26	25.5 (25.0 ÷ 26.0)		

*: Statistical significant; **: Highly statistical significant.

**Table 11:** Primary and secondary scores in patients eligible for acrylic prostheses.

ACRYLIC PROSTHESIS SCORES		Low		Medium		Good		Very Good		Marginal Homogeneity Test
		*n*	%	*n*	%	*n*	%	*n*	%	
Dental support	Primary					2	100.0			-
	Secondary					2	100.0			
Periodontal support	Primary							2	100.0	-
	Secondary							2	100.0	
Mucosal support	Primary			2	100.0					-
	Secondary			2	100.0					
Bone support	Primary					2	100.0			-
	Secondary					2	100.0			
Occlusion	Primary	2	100.0							-
	Secondary							2	100.0	
Mandibulo-cranial relationships	Primary	2	100.0							-
	Secondary							2	100.0	
TOTAL SCORE	Primary					2	100.0			-
	Secondary							2	100.0	

**Table 12:** Primary and secondary scores in patients eligible for composite prostheses with rigid SRMs.

COMPOSITE PROSTHESIS WITH RIGID SRM SCORES		Low		Medium		Good		Very Good		Marginal Homogeneity Test
		*n*	%	*n*	%	*n*	%	*n*	%	
Dental support	Primary			11	42.3	11	42.3	4	15.4	*p* < 0.001 **
	Secondary			2	7.7	10	38.5	14	53.8	
Periodontal support	Primary					16	61.5	10	38.5	-
	Secondary							26	100.0	
Mucosal support	Primary			5	19.2	17	65.4	4	15.4	*p* = 0.025 *
	Secondary					22	84.6	4	15.4	
Bone support	Primary			7	26.9	12	46.2	7	26.9	*p* = 0.013 *
	Secondary			2	7.7	13	50.0	11	42.3	
Occlusion	Primary	23	88.5			3	11.5			*p* < 0.001 **
	Secondary	12	46.2			2	7.7	12	46.2	
Mandibulo-cranial relationships	Primary	9	34.6	6	23.1	5	19.2	6	23.1	*p* = 0.041 *
	Secondary	4	15.4	8	30.8	5	19.2	9	34.6	
TOTAL SCORE	Primary			5	19.2	21	80.8			*p* = 0.001 **
	Secondary			2	7.7	12	46.2	12	46.2	

*: Statistical significant; **: Highly statistical significant.

**Table 13:** Primary and secondary scores in patients eligible for composite prostheses with semi-rigid SRMs.

COMPOSITE PROSTHESIS WITH SEMI-RIGID SRM SCORES		Low		Medium		Good		Very Good		Marginal Homogeneity Test
		*n*	%	*n*	%	*n*	%	*n*	%	
Dental support	Primary			6	66.7	3	33.3			*p* = 0.014 *
	Secondary			3	33.3	3	33.3	3	33.3	
Periodontal support	Primary			3	33.3	3	33.3	3	33.3	*p* = 0.083
	Secondary					6	66.7	3	33.3	
Mucosal support	Primary			3	33.3	6	66.7			-
	Secondary					9	100.0			
Bone support	Primary			3	33.3	6	66.7			-
	Secondary					9	100.0			
Occlusion	Primary	9	100.0							*p* = 0.083
	Secondary	6	66.7					3	33.3	
Mandibulo-cranial relationships	Primary	9	100.0							*p* = 0.028 *
	Secondary	3	33.3	3	33.3			3	33.3	
TOTAL SCORE	Primary			9	100.0					*p* = 0.020 *
	Secondary			3	33.3	3	33.3	3	33.3	

*: Statistical significant.

**Table 14:** Primary and secondary scores in patients eligible for elastic prostheses.

ELASTIC PROSTHESIS SCORES		Low		Medium		Good		Very Good		Marginal Homogeneity Test
		*n*	%	*n*	%	*n*	%	*n*	%	
General status	Primary					6	16.7	30	83.3	*p* = 0.083
	Secondary					3	8.3	33	91.7	
Dental support	Primary			9	25.0	24	66.7	3	8.3	*p* < 0.001 **
	Secondary			3	8.3	18	50.0	15	41.7	
Periodontal support	Primary			3	8.3	6	16.7	27	75.0	*p* = 0.014 *
	Secondary					6	16.7	30	83.3	
Mucosal support	Primary					24	66.7	12	33.3	*p* = 1.000
	Secondary					24	66.7	12	33.3	
Bone support	Primary			3	8.3	18	50.0	15	41.7	*p* = 0.083
	Secondary			3	8.3	15	41.7	18	50.0	
Occlusion	Primary	27	75.0	3	8.3	6	16.7			*p* < 0.001 **
	Secondary	15	41.7	3	8.3	9	25.0	9	25.0	
Mandibulo-cranial relationships	Primary	18	50.0	3	8.3	3	8.3	12	33.3	*p* = 0.003 **
	Secondary	9	25.0	3	8.3	3	8.3	21	58.3	
TOTAL SCORE	Primary			6	16.7	27	75.0	3	8.3	*p* = 0.001 **
	Secondary			3	8.3	18	50.0	15	41.7	

*: Statistical significant; **: Highly statistical significant.

**Table 15:** Primary and secondary scores in patients eligible for clasp-retained skeletal prostheses.

CLASP-RETAINED SKELETAL PROSTHESIS SCORES		Low		Medium		Good		Very Good		Marginal Homogeneity Test
		*n*	%	*n*	%	*n*	%	*n*	%	
General status	Primary					3	4.1	70	95.9	*p* = 1.000
	Secondary					3	4.1	70	95.9	
Dental support	Primary			23	31.5	43	58.9	7	9.6	*p* < 0.001 **
	Secondary			10	13.7	31	42.5	32	43.8	
Periodontal support	Primary			4	5.5	26	35.6	43	58.9	*p* < 0.001 **
	Secondary					13	17.8	60	82.2	
Mucosal support	Primary			6	8.2	51	69.9	16	21.9	*p* < 0.001 **
	Secondary					50	68.5	23	31.5	
Bone support	Primary			3	4.1	32	43.8	38	52.1	*p* = 0.083
	Secondary			3	4.1	29	39.7	41	56.2	
Occlusion	Primary	60	82.2	7	9.6	3	4.1	3	4.1	*p* < 0.001 **
	Secondary	38	52.1	3	4.1	13	17.8	19	26.0	
Mandibulo-cranial relationships	Primary	38	52.1	3	4.1	7	9.6	25	34.2	*p* < 0.001 **
	Secondary	23	31.5	3	4.1	6	8.2	41	56.2	
TOTAL SCORE	Primary			18	24.7	49	67.1	6	8.2	*p* < 0.001 **
	Secondary			6	8.2	38	52.1	29	39.7	

**: Highly statistical significant.

**Table 16:** Primary and secondary scores in patients eligible for fixed implant-prosthetic rehabilitation.

FIXED IMPLANT-PROSTHETIC REHABILITATION SCORES		Low		Medium		Good		Very Good		Marginal Homogeneity Test
		*n*	%	*n*	%	*n*	%	*n*	%	
Dental support	Primary			6	14.3	30	71.4	6	14.3	*p* < 0.001 **
	Secondary					24	57.1	18	42.9	
Periodontal support	Primary			6	14.3	12	28.6	24	57.1	*p* < 0.001 **
	Secondary			3	7.1	6	14.3	33	78.6	
Mucosal support	Primary			6	14.3	15	35.7	21	50.0	*p* = 0.005 **
	Secondary					15	35.7	27	64.3	
Bone support	Primary			6	14.3	24	57.1	12	28.6	*p* = 0.014 *
	Secondary			6	14.3	18	42.9	18	42.9	
Occlusion	Primary	36	85.7	3	7.1	3	7.1			*p* < 0.001 **
	Secondary	15	35.7	3	7.1	6	14.3	18	42.9	
Mandibulo-cranial relationships	Primary	15	35.7	3	7.1	6	14.3	18	42.9	*p* < 0.001 **
	Secondary	6	14.3	3	7.1	3	7.1	30	71.4	
TOTAL SCORE	Primary			3	7.1	33	78.6	6	14.3	*p* < 0.001 **
	Secondary					21	50.0	21	50.0	

*: Statistical significant; **: Highly statistical significant.

**Table 17:** Primary and secondary scores in patients eligible for hybrid removable implant-prosthetic rehabilitation.

REMOVABLE HYBRID IMPLANT-PROSTHETIC REHABILITATION SCORES		Low		Medium		Good		Very Good		Marginal Homogeneity Test
		*n*	%	*n*	%	*n*	%	*n*	%	
Dental support	Primary					6	100.0			-
	Secondary							6	100.0	
Periodontal support	Primary					3	50.0	3	50.0	-
	Secondary							6	100.0	
Mucosal support	Primary					3	50.0	3	50.0	*p* = 1.000
	Secondary					3	50.0	3	50.0	
Bone support	Primary			3	50.0			3	50.0	*p* = 1.000
	Secondary			3	50.0			3	50.0	
Occlusion	Primary	6	100.0							*p* = 0.028 *
	Secondary			3	50.0			3	50.0	
Mandibulo-cranial relationships	Primary	3	50.0					3	50.0	-
	Secondary							6	100.0	
TOTAL SCORE	Primary					3	50.0	3	50.0	-
	Secondary							6	100.0	

*: Statistical significant.

## Data Availability

The original contributions presented in this study are included in the article.

## Abbreviations

The following abbreviations are used in this manuscript:

MC	maxillo-cranial
Md	mandibular
Mx	maxillary
SSDS	stomatognathic system disfunctional syndrome
TMJ	temporo-mandibular joint
